# Development of a Large-Scale Roadside Facility Detection Model Based on the Mapillary Dataset

**DOI:** 10.3390/s22249992

**Published:** 2022-12-19

**Authors:** Zhehui Yang, Chenbo Zhao, Hiroya Maeda, Yoshihide Sekimoto

**Affiliations:** 1 Center for Spatial Information Science, The University of Tokyo, Tokyo 277-8568, Japan; 2 Urban X Technologies, Shibuya-ku, Tokyo 150-0002, Japan

**Keywords:** object detection, YOLOv7, YOLOx, Mask R-CNN, ITS, HD map

## Abstract

The detection of road facilities or roadside structures is essential for high-definition (HD) maps and intelligent transportation systems (ITSs). With the rapid development of deep-learning algorithms in recent years, deep-learning-based object detection techniques have provided more accurate and efficient performance, and have become an essential tool for HD map reconstruction and advanced driver-assistance systems (ADASs). Therefore, the performance evaluation and comparison of the latest deep-learning algorithms in this field is indispensable. However, most existing works in this area limit their focus to the detection of individual targets, such as vehicles or pedestrians and traffic signs, from driving view images. In this study, we present a systematic comparison of three recent algorithms for large-scale multi-class road facility detection, namely Mask R-CNN, YOLOx, and YOLOv7, on the Mapillary dataset. The experimental results are evaluated according to the recall, precision, mean F1-score and computational consumption. YOLOv7 outperforms the other two networks in road facility detection, with a precision and recall of 87.57% and 72.60%, respectively. Furthermore, we test the model performance on our custom dataset obtained from the Japanese road environment. The results demonstrate that models trained on the Mapillary dataset exhibit sufficient generalization ability. The comparison presented in this study aids in understanding the strengths and limitations of the latest networks in multiclass object detection on large-scale street-level datasets.

## 1. Introduction

Object detection is considered the first stage in the high-definition (HD) map construction framework. It also plays an essential role in the field of autonomous driving. Moreover, the detection and monitoring of road facilities and infrastructures are essential for HD map construction in the autonomous driving field, and for intelligent transportation systems (ITSs). Traditional techniques for HD map construction and ADAS development require costly devices [1] such as IMUs, LiDAR sensors, and ranging sensors, which are time consuming for HD map processing. Furthermore, these shortcomings make it difficult to achieve the real-time updation of HD maps and low processing cost. Hence, vision-based deep-learning methods for HD map construction in ADASs have become feasible, and have been the subject of active investigation.

The on-board camera is a common sensor used extensively in autonomous driving. In recent years, the convolutional neural network (CNN), which is an innovative computer vision technique, has been successfully applied and combined with images captured by on-board cameras to realize HD map reconstruction. Many studies have focused on low-cost HD mapping using lightweight camera sensors. Such vision-based deep-learning methods rely on cameras that are mounted on vehicles to record continuous images, and they extract the mapping information from the images to the cloud for map processing.

Road facilities and roadside infrastructures (e.g., traffic signs, traffic lights, road lights, guard rails, electric poles) are fundamental elements in HD map construction. The complete and successful detection of these road elements from images can provide rich road information and ensure safety in autonomous driving. Some researchers have performed traffic sign detection [2] and traffic light detection [3], improving YOLOv4 for driverless cars. However, their methods and targets are limited to a single-class road object without paying attention to the detection performance of the model for multi-class road objects. However, as real-world road conditions and environment are complex and change constantly, autonomous driving and road HD maps require accuracy detection and real-time updates of road objects. Therefore, various vision-based deep-learning algorithms have been proposed for real-time image processing in updating HD maps. Some recent research pay great attention on developing the optimization algorithms in related fields as well [4,5]. The validation of the performance and feasibility of new state-of-the-art deep-learning methods is essential pre-exploration work.

**Figure 1:**
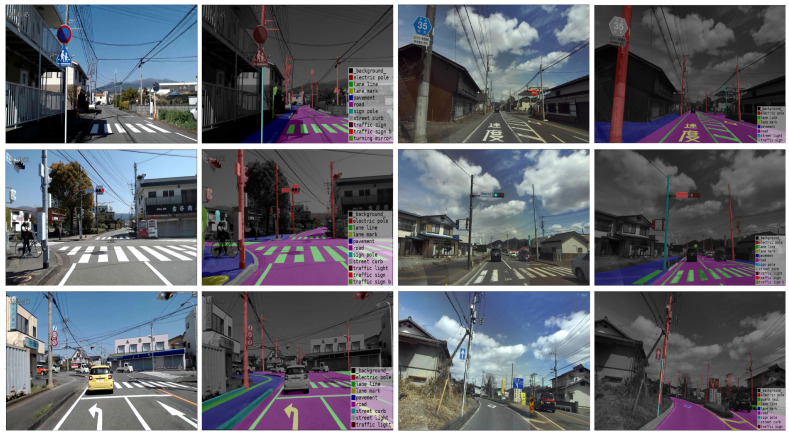
Sample annotation of Japanese road-view dataset.

In this study, we aimed to verify the performances of the most recent deep-learning algorithms in large-scale road facility detection using a benchmark street-level dataset. Our key contributions are summarized as follows:

- Training experiments were carried out on three state-of-the-art object detection algorithms for multi-class road facility and infrastructure detection, namely Mask R-CNN, YOLOx, and YOLOv7, using the transferred Mapillary street-level dataset, which contains complex weather conditions as well as different environments.
- The comparative evaluation results of the algorithms were compared and summarized to demonstrate the performance of each model according to several metrics, including the recall, precision, memory usage, and number of FLOPS.
- A finely annotated semantic segmentation road facility dataset was developed, which was used to verify the generalization capability of the road facility detection performance of the developed models. Figure 1 presents several examples of our annotated images.
- The experimental results have the potential to enhance the understanding of researchers and practitioners, and provide a preview of the performance of the latest detection models in complex road environments.

The remainder of this paper is organized as follows: Section 2 reviews the related works. Section 3 introduces the concepts of the deep-learning methods used in this work. The experiments on our custom dataset and the Mapillary Vistas dataset for model training are explained in Section 4. The results and a discussion are presented in Section 5. Finally, Section 6 concludes the paper.

## 2. Related Work

In this section, studies on road object detection algorithms are introduced and their relationship with HD mapping is discussed.

### 2.1. Road Object Detection

Object detection in road spaces is a challenging core task in many vision-based applications. In particular, many vision-based deep-learning detection methods have been applied in the autonomous driving field to advance driving and mapping technologies. The main tasks include landmark detection, pedestrian detection, vehicle detection, traffic sign and traffic light recognition, roadside infrastructure detection, and traffic object counting.

The work in [6] used the KITTI database and provided a subject analysis on several mainstream deep-learning algorithms for vehicle and pedestrian detection. However, this work did not consider algorithm training in different weather and environmental conditions, and failed to investigate the most recent algorithms, as deep-learning methods are updated quite rapidly. Algorithms that are trained on richly diverse datasets tend to exhibit superior performance and practical value in real-world applications. There are some proposals [7,8] which accurately reconstructed landmarks on road surfaces based on plane assumption with deep learning methods. Authors in another study focused on pedestrian detection only, and developed a two-stage object detector [9]. The detection and recognition of traffic signs, which are one of the most important components in traffic systems, is an active research topic. The authors of [10,11] conducted intensive work on traffic sign detection and classification based on deep-learning methods. Moreover, they developed large-scale traffic sign datasets in Europe and China, respectively. Ashutosh et al. [12] used YOLOv4 [13] for vehicle detection and traffic flow estimation. In [14], an analysis survey on the vehicle detection of several state-of-the-art algorithms was conducted. However, previous works did not pay great attention to multi-category road objects. Hence, the continual evaluation and comparison of new algorithms on different tasks is important.

### 2.2. Road Object Detection for HD Mapping

HD maps play a key role in the development of autonomous driving. An HD map provides high-precision road space object information regarding the real-world driving environment. Traditional methods for constructing high-accuracy maps use expensive and laborious LiDAR equipment to extract point clouds of the environment [15]. The whole map reconstruction pipeline requires a large number of professional mapping vehicles to generate timely mapping information. Furthermore, manual or semi-manual automated work is required to construct HD maps, which necessitates a huge amount of manpower and software efforts [16,17].

In recent years, increasing attention has been paid to low-cost and light-weight mapping techniques. An increasing number of vision-based methods using deep learning have been developed with improved accuracy compared to previous radar-based methods. Deep-learning techniques have emerged as an effective tool for HD map construction. In particular, increasing efforts have been made in mobile motor industries (such as Tesla and Waymo) to develop vision-based HD map construction for vehicles using low-cost camera sensors. Object detection algorithms are regarded as the optimal solution for object perception in HD map reconstruction. Yao et al. proposed MVSNet to extend the cost volume for depth estimation from multiple images [18]. Several previous works focused on lane lines and marks on the road surface, and they used plane assumption to locate the position [8]. Another recent study [19] presented a new method for traffic sign and roadside pole reconstruction in HD maps. The roadside object detection was performed by deep-learning-based object detectors. Road space facilities and road objects in HD map reconstruction have been studied extensively based on deep-learning object detectors and techniques.

In [20], a new system to detect road changes for HD map updation was proposed. Deep-learning-based systems for HD maps have been researched and developed extensively in recent years. However, road object detection remains a challenging core task in many vision-based applications, especially in the advancement of driving and mapping technologies.

## 3. Methodology

In recent years, object detection algorithms in deep learning have been updated and iterated with relatively high frequency. Thus, the state-of-the-art algorithms are more informative for conducting experiments. Object detection methods can be categorized into two classes: one-stage detectors and two-stage detectors. In this work, we adopted Mask R-CNN [21] from among the two-stage detectors. YOLOx and YOLOv7 were selected from among the one-stage detectors.They are all state-of-the-art algorithms in each category. We present the details of these mainstream networks for road facility detection in this section.

### 3.1. Two-Stage Object Detection Networks

Two-stage networks are classified as region-based methods [22], which exhibit higher accuracy for object detection but are slower than one-stage detectors.

Mask R-CNN: He et al. proposed the mask region-based CNN (Mask R-CNN) algorithm. Mask R-CNN is an extension of Faster R-CNN [23] for segmentation, both of which comprise two modules. The first stage is the region proposal network (RPN), which is a deep convolutional network. The RPN takes the input image and creates a region proposal of the possible positions of objects using the objectness score. The second stage is known as the region-based CNN, in which Fast R-CNN classifies the objects according to the features extracted from the proposed region and performs a regression of the bounding box. Mask R-CNN improves the performance of the system for small object detection in Faster R-CNN by combining the backbone network with a feature pyramid network [10,24]. The backbone network architecture of Faster R-CNN is VGG16 [25], which is substituted with a residual network (ResNet) [26] in Mask R-CNN. By adopting an end-to-end learning method, Mask R-CNN can be trained for two tasks: object detection, which generates a bounding box, and pixel-level segmentation for the detected objects.

**Figure 2:**
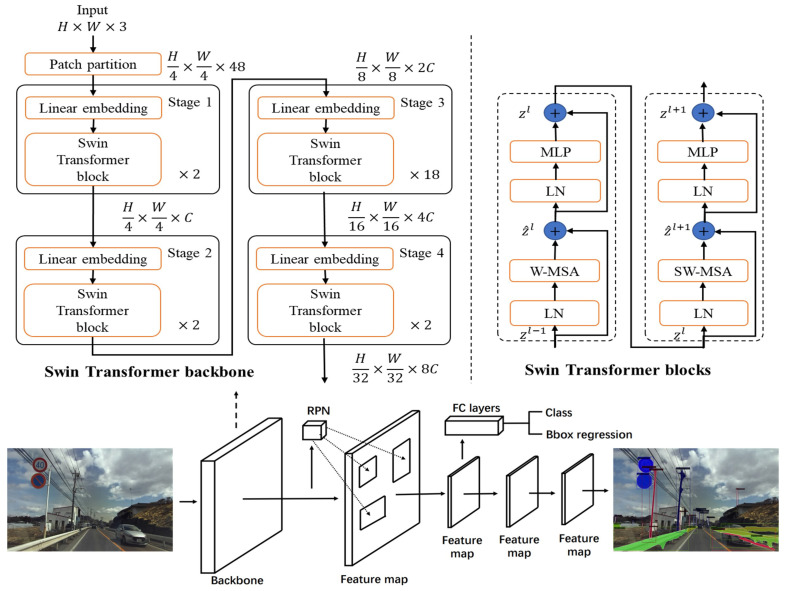
Architecture of Mask R-CNN with Swin Transformer.

Although target detection is not the main task of Mask R-CNN, it still exhibits the best detection performance among the two-stage detection algorithms. In this study, we replaced ResNet-101 with the Swin Transformer [27] as the backbone in Mask R-CNN on the Mapillary Vistas dataset [28]. The Swin Transformer is a state-of-the-art feature extractor with a self-attention mechanism. The Swin Transformer is highly efficient, and exhibits higher accuracy than ResNet-101. It is used as the backbone in many vision-based algorithm architectures for feature extractors owing to its desirable performance and properties. Figure 2 depicts the Swin Transformer architecture and overall network structure of Mask R-CNN.

### 3.2. One-Stage Object Detection Networks

The most significant difference between the one-stage and two-stage object detection networks is that there is no region proposal step and the detected bounding boxes are directly determined from the input image in a one-stage network. Among representative one-stage detectors, the You Only Look Once (YOLO) algorithms have balanced high speed and accuracy for object detection tasks. The first three generations achieved varying results, with unsatisfactory performance on small objects [29], low recall [30], and limitations on bounding box predictions [30]. In 2020, Bochkovskiy et al. proposed YOLOv4 [13], which exhibited significant improvements over the previous versions. A new feature extractor known as CSPDarknet-53 enhances the learning capacity of the network and reduces the memory cost in YOLOv4. Owing to various innovative enhancements, YOLOv4 can achieve excellent performance in real-time processing.

Although YOLOv5 was released in 2020, it is difficult for this network to detect small objects, such as traffic signs and traffic lights in complex road conditions [31]. However, the YOLO series are updated quite frequently. In this research, we tested the two latest algorithms in the YOLO series.

YOLOx: Ge et al. released this state-of-the-art object detection model in August 2021 [32], which is an anchor-free version of the conventional YOLO. They introduced the decoupled head and leading label assignment strategy, SimOTA, to achieve superior performance over YOLOv5. YOLOx has achieved the same accuracy as YOLOv4 with half of the inference time, according to measurements on COCO [33].

YOLOv7: As the newest version in the YOLO series, YOLOv7 [34] has achieved the best mAP on the COCO benchmark dataset. Furthermore, it exhibits a significantly more rapid inference time compared to its predecessors, owing to several new trainable bag-of-freebies methods. It has exceeded all existing object detection algorithms in terms of both speed and accuracy in the range of 5–160 FPS, and has achieved a 56.8% average precision, which is the best score among all known real-time object detectors.

## 4. Experiments

### 4.1. Dataset Preparation

#### 4.1.1. Custom Dataset

We developed a novel finely annotated Japanese driving segmentation dataset for the model testing of road facility detection. Our testing dataset was obtained from two sources. We selected and extracted 700 frames from the vehicle orientation dataset [12] developed by Ashutosh et al. in 2021 and 500 high-quality driving images from our driving experiments, which were conducted in a Japanese rural area using a ZED camera.

The selected images were all obtained from a forward-looking driving view in urban and rural areas of Japan. All images had a wide-angle view, and they covered all types of road facilities and roadside infrastructures of Japan. We provided dense and fine-grained semantic annotations using polygons for all individual road facilities in all images.

#### 4.1.2. Mapillary Vistas Dataset

This dataset is a novel large-scale street-view benchmark image dataset that contains 25,000 high-resolution images annotated into 66/123 object classes, of which 37/70 classes are instance segmentation annotations (v.1.2 and v.2.0, respectively). As the images in this dataset were captured globally in various conditions relating to weather, season, and daytime, models that are trained with this dataset exhibit good generalization performance on similar road-view datasets. In this work, we used v.2.0 for the experiments.

There exist other publicly available standard datasets, such as KITTI [35] and BDD100K [36], which were captured from street views. However, the images in the KITTI dataset mainly originate from the European region and were captured on sunny days or during the daytime. The BDD100K dataset contains more complex weather conditions, but the data were all collected from the United States. Thus, deep-learning models that are trained with these datasets lack generalization abilities for other testing datasets.

The entire Mapillary dataset was divided into training, validation, and test sets. The training and validation datasets contained 18,000 and 2000 images with annotation, respectively. Labels were withheld for 5000 images in the test set. Therefore, we used the validation set to evaluate the model performance, as the validation images would not be used for the model learning during the training process.

### 4.2. Implementation Details

We performed training on the Mapillary Vistas dataset for Mask R-CNN, YOLOx, and YOLOv7. The model evaluation was conducted on the test set of the Mapillary Vistas dataset. Subsequently, we performed an extensive evaluation on our custom dataset.

The popular object detection toolbox and benchmark MMDetection [37] provides an open-source platform for implementing the most recent deep-learning algorithms based on PyTorch. The decomposed detection framework can be easily constructed using a different customized object detection framework.

**Figure 3:**
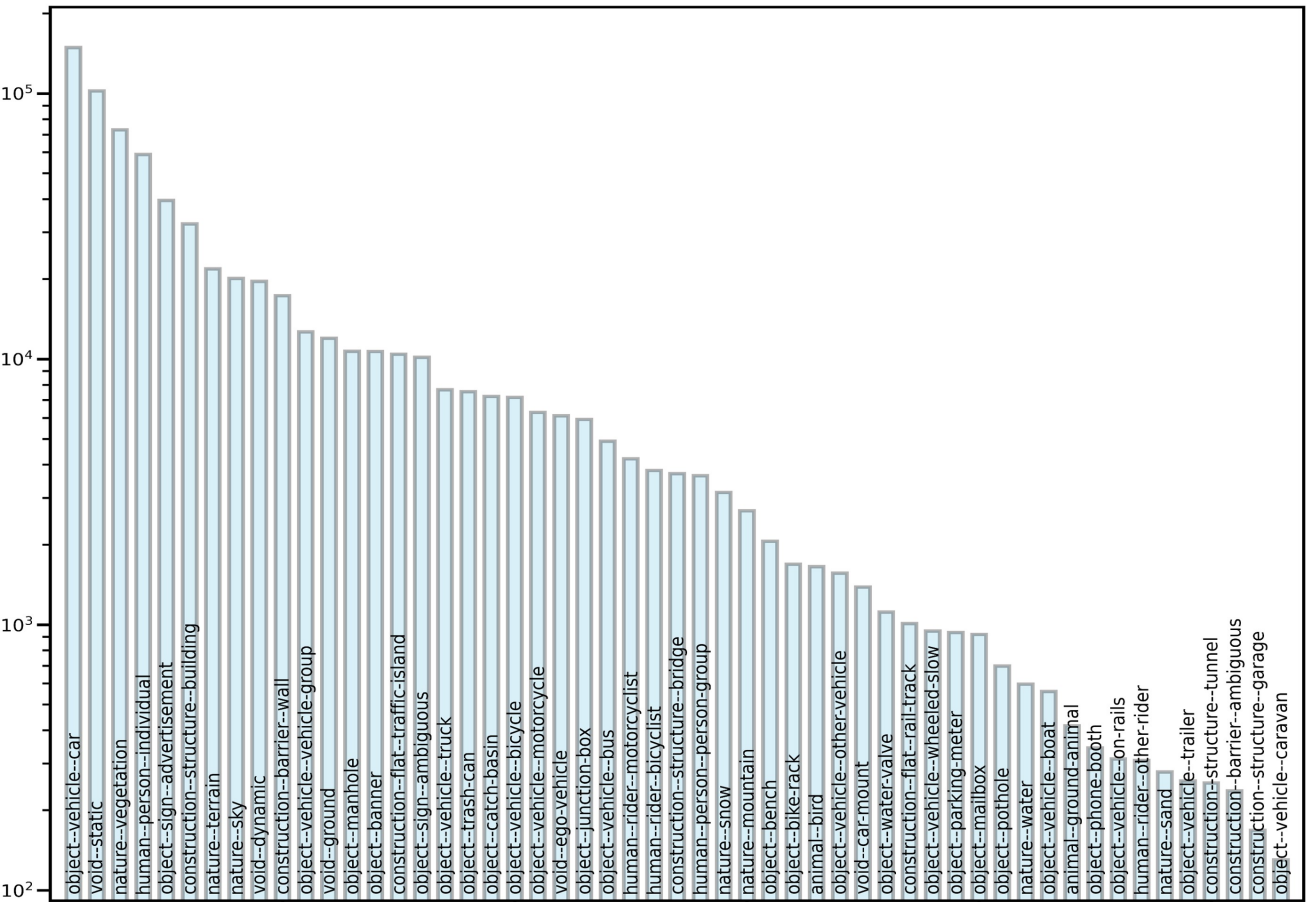
Illustration of removed classes.

**Figure 4:**
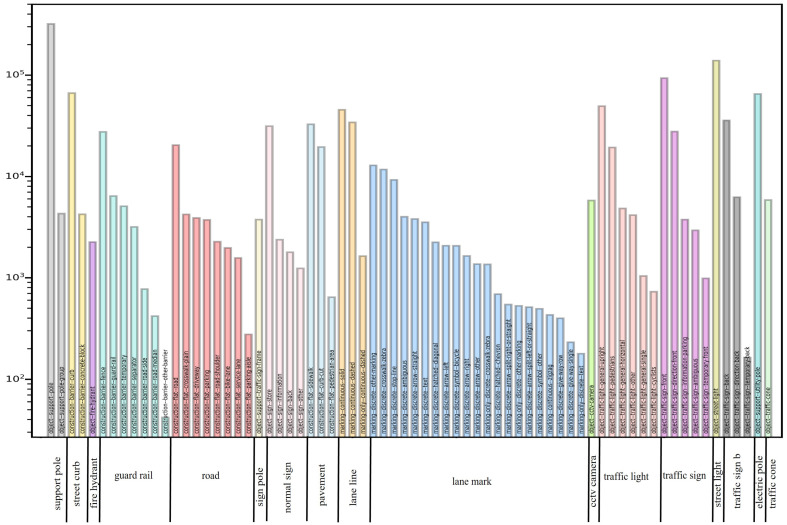
Number of labeled instances per class and class merging.

We first removed 52 classes that did not belong to our detection target from the original labeled JSON file in the dataset to develop a detection model that only detected road facilities and roadside infrastructures. Following this step, 71 classes remained; to ensure that the trained model was close to our custom dataset that we used for testing, we merged these 71 categories into 17 classes. We first removed the categories “Lane line” and “Lane mark”, which are not road appendages, from the merged dataset. Given our previous experimental results, we also removed the categories “Road” and “Pavement” from the merged dataset, because the detection frames generated in the images were large in proportion to the images, and contained many noise points. Finally, 13 classes of road facilities remained in the entire dataset. Figure 3 depicts the removed classes, and the detailed information of the merging is illustrated in Figure 4.

We maintained the same volume as the original Mapillary dataset for the training phase. Therefore, the training and validation datasets consisted of 18,000 and 2000 images with 13 road facility classes, respectively. The hyperparameters setting in the training of each model is based on the setting in the respective original paper. Batch size is optimally set with the memory of GPUs. The maximum learning rate after warming up is set with the batch size by following the linear growth rule. The hyperparameter settings for the training of each model are shown in detail as follows:

1. Mask R-CNN: We trained Mask R-CNN using the MMdetection platform on four NVIDIA A100 Tensor Core GPUs (NVIDIA, CA, USA) for 20 epochs and batch = 24, with the model pretrained using the ImageNet [38] dataset. The initial learning rate was set to 0.001 and warmed up at 1000 iterations. The optimizer was AdamW [39].
2. YOLOx: This model was also trained on four NVIDIA A100 Tensor Core GPUs. We increased the training epochs up to 300 with batch = 64. The model was pretrained on the COCO [33] dataset. The initial learning rate was set to 0.01 and warmed up at 5 epochs. The optimizer for the training was SGD.
3. YOLOv7: As YOLOv7 was only released recently, we trained the model using the Mapillary dataset from the official GitHub on the same machine. The initial learning rate was 0.01 and warmed up at 3 epochs; training was performed for 300 epochs, and batch = 64. The model was pretrained on the COCO dataset and the optimizer was SGD.

## 5. Results

In this section, we demonstrate the performance of the road facility detection models that were trained on the transferred Mapillary dataset, as described above. All models were evaluated according to the precision, recall, and F1-score in terms of bounding box. Furthermore, we evaluated the object detection models using additional metrics, such as the parameter size and GFLOPS. We tested all models on NVIDIA A100 Tensor Core GPUs with 40 GB of GPU memory.

### 5.1. Evaluation Metrics and Results

Recall, precision, and F1-score are evaluation metrics that can be used for the detection phase. The F1-score combines the precision and recall into a single metric, and is calculated using (3).

(1) $$\;\mathrm{Recall}=\frac{\mathrm{TP}}{\mathrm{TP}+\mathrm{FN}}=\frac{\mathrm{TP}}{\mathrm{all}\;\mathrm{detected}\;\mathrm{object}},$$

(2) $$\mathrm{Precision}=\frac{\mathrm{TP}}{\mathrm{TP}+\mathrm{FP}}=\frac{\mathrm{TP}}{\mathrm{all}\;\mathrm{ground}\;\mathrm{truth}},$$

(3) $$F1=2\times \frac{\mathrm{precision}\times \mathrm{recall}}{\mathrm{precision}+\mathrm{recall}},$$

where true positive (TP) indicates that the detected bounding box has an IoU > 45% and the predicted class is the same as the ground truth. False positive (FP) and false negative (FN) indicate the numbers of detections in the results and in the ground truth, respectively.

**Figure 5:**
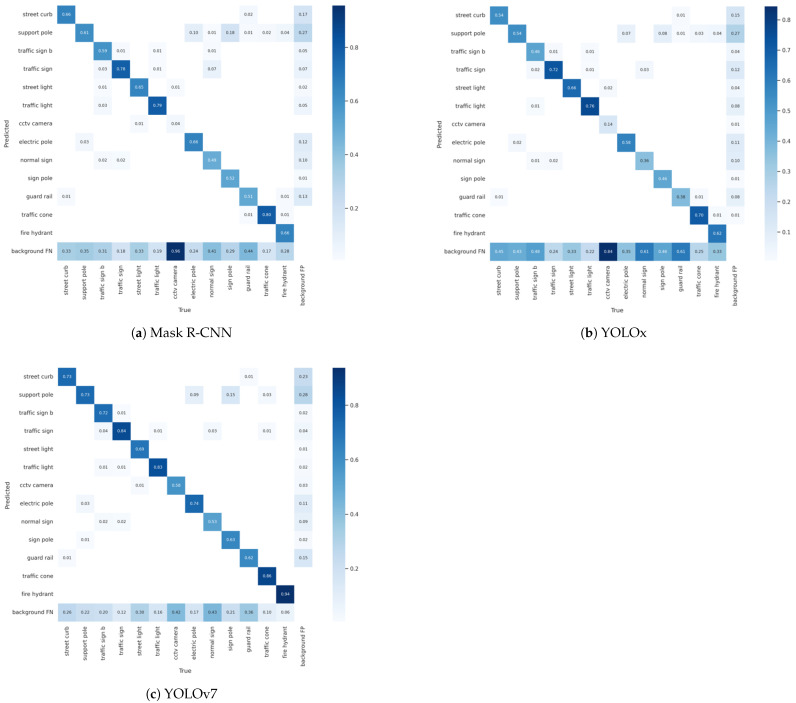
Normalized confusion matrices of detection results.

A confusion matrix provides an easy means of visualizing and summarizing the model classification performance. We used normalized confusion matrices to present the detailed comparison and detection results of each class. Figure 5 depicts the confusion matrices of the detection models, which provide more information on the FN and FP than a visual observation of the test results.

Table 1 displays the evaluation metrics of the road facility detection models on the Mapillary dataset with IoU = 0.45.

Table 2 lists the memory size and computation of each model. The GFLOPS provide a platform-independent metric for measuring the computational efficiency of a model. The size of the model was calculated as the total number of parameters with intermediate activation maps [31].

**Figure 6:**
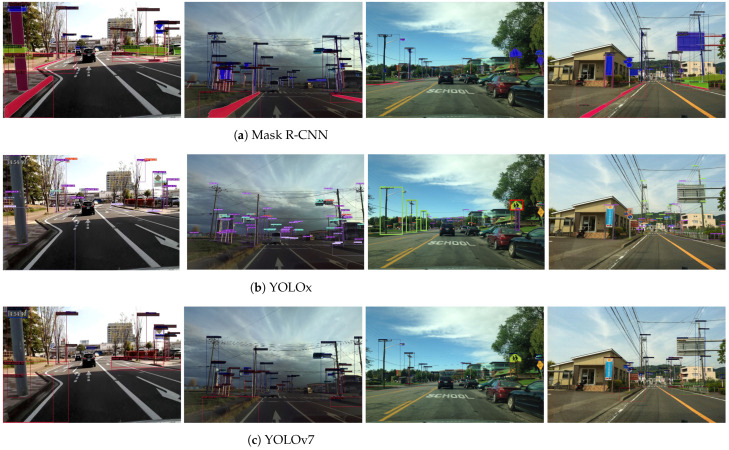
Qualitative comparsion of output from different models on custom dataset and Mapillary dataset.

We also validated the trained models on our custom dataset to validate their generalization ability. Figure 6 presents the qualitative comparison results of the outputs of the three models. The two left columns represent our custom dataset and the two right columns represent the Mapillary Vistas validation dataset.

### 5.2. Discussion

The YOLOv7 detection model achieved higher precision and recall for road facility and roadside infrastructure detection than Mask R-CNN and YOLOx, even when it was trained with a different dataset. Thus, it can provide robust detection performance on other road-view images that are collected using different devices and in varying regions.

Owing to its balanced model size and computation, YOLOv7 exhibited excellent performance. Although the number of model parameters of YOLOv7 was approximately three times that of YOLOx, it achieved higher accuracy in road facility detection, and it was only 1.3 times more computationally intensive than YOLOx. YOLOv7 exhibited better detection performance when the number of parameters was close to that of Mask R-CNN, and the computation of Mask R-CNN was eight times that of YOLOv7. It is known that two-stage detection networks achieve better detection performance than one-stage detection networks, but YOLOv7, which is the latest version in the YOLO series, achieved superior performance in roadside infrastructure detection with a balanced speed and model size.

The qualitatively evaluated results on our custom dataset demonstrated that the models that were trained on the Mapillary Vistas dataset exhibited a good generalization capacity on random street-view images in terms of road facility detection. Furthermore, as the training images in the Mapillary dataset have strong diversity, models that are trained on this dataset will have a sufficient generalization ability on other similar datasets.

## 6. Conclusions

The detection of road facilities and roadside infrastructure is an indispensable task in ITSs and HD mapping. In this study, we conducted systematic and experimental comparisons among Mask R-CNN, YOLOx, and YOLOv7, which are three recent objection detection algorithms, on the large-scale street-level Mapillary Vistas dataset in terms of road facility detection. YOLOv7 achieved the best detection performance. Furthermore, we tested the model performance on our custom finely annotated Japanese driving-view road facility dataset. The results demonstrate that object detectors trained on the Mapillary Vistas dataset offer a strong generalization ability.

Although object detection algorithms have diverse applications, based on our experimental results, our work can be improved to provide a better illustration of the latest deep-learning algorithms for road facility detection to contribute to HD map construction and ITS. With the rapid developments in the field of deep-learning-based ITS applications, state-of-the-art deep-learning algorithms for road space object detection require further intensive study in the future.

## Figures and Tables

**Table 1:** Detection results (IoU threshold = 45%).

Network	Recall	Precision	F1-Score
Mask R-CNN	59.77%	82.96%	67.13%
YOLOx	54.25%	84.33%	63.25%
YOLOv7	**72.60%**	**87.57%**	**78.70%**

**Table 2:** Summary of memory and computation of models.

Network	Parameters (m)	GFLOPS
Mask R-CNN	107	838
YOLOx	25	74
YOLOv7	82	102

## Data Availability

Not applicable.
